# Acceptance of a French e–Mental Health Information Website (CléPsy) for Families: A Web-Based Survey

**DOI:** 10.2196/50978

**Published:** 2024-08-15

**Authors:** Benjamin Landman, Elie Khoury, Alicia Cohen, Vincent Trebossen, Alexandre Michel, Aline Lefebvre, Richard Delorme

**Affiliations:** 1 Child and Adolescent Psychiatry Department, Robert Debré Hospital, Assistance Publique - Hopitaux de Paris, 48 Boulevard Serurier, Paris, 75019, France, 33 185552762; 2 Paris University, Paris, France; 3 Centre Hospitalier Spécialisée Fondation Vallee, Gentilly, France; 4 Human Genetics and Cognitive Functions, Institut Pasteur, Paris, France

**Keywords:** mental health education, children, family, child, pediatrics, pediatric, mental health, parent, parents, parenting, psychiatry, website, acceptance, patient education, online information, health information, ease of use, usefulness, survey, surveys, user, experience, questionnaire, questionnaires, families

## Abstract

**Background:**

Childhood mental health issues concern a large amount of children worldwide and represent a major public health challenge. The lack of knowledge among parents and caregivers in this area hinders effective management. Empowering families enhances their ability to address their children’s difficulties, boosts health literacy, and promotes positive changes. However, seeking reliable mental health information remains challenging due to fear, stigma, and mistrust of the sources of information.

**Objective:**

This study evaluates the acceptance of a website, CléPsy, designed to provide reliable information and practical tools for families concerned about child mental health and parenting.

**Methods:**

This study examines user characteristics and assesses ease of use, usefulness, trustworthiness, and attitude toward using the website. Platform users were given access to a self-administered questionnaire by means of mailing lists, social networks, and posters between May and July 2022.

**Results:**

Findings indicate that the wide majority of the 317 responders agreed or somewhat agreed that the website made discussions about mental health easier with professionals (n=264, 83.3%) or with their relatives (n=260, 82.1%). According to the ANOVA, there was a significant effect between educational level and perceived trust (*F*_6_=3.03; *P*=.007) and between frequency of use and perceived usefulness (*F*_2_=4.85; *P*=.008).

**Conclusions:**

The study underlines the importance of user experience and design in web-based health information dissemination and emphasizes the need for accessible and evidence-based information. Although the study has limitations, it provides preliminary support for the acceptability and usefulness of the website. Future efforts should focus on inclusive co-construction with users and addressing the information needs of families from diverse cultural and educational backgrounds.

## Introduction

Childhood mental health (MH) issues concern up to 13.4% of children worldwide [1] and represent a major public health challenge [2]. However, parents’ and caregivers’ lack of knowledge in this area constitutes a major obstacle to their management of these issues [3]. Child and adolescent MH has substantially worsened in recent years, especially during the COVID-19 pandemic, notably in girls and vulnerable populations such as families with low income or children and adolescents with pre-existing MH difficulties [4].

The World Health Organization has promoted a shift from patient-centered health to family empowerment as a catalyst for health development especially in chronic diseases. This switch involved providing families with tools to understand their rights regarding health care and how to access health services [5]. For example, patient empowerment is known to enhance health literacy in people with diabetes through increasing expression, self-judgment, and critical thinking, especially communicative health literacy and critical health literacy that relies on interaction and communication skills [6]. Parent health literacy was also considered an essential element in minimizing childhood obesity in a meta-analysis [7]. In the specific case of MH, family empowerment is a mediator for positive changes by enabling better management of children’s difficulties and providing a greater sense of confidence in parental problem-solving abilities, facilitating the resolution of future MH challenges [8].

Health literacy encompasses knowledge that helps individuals achieve and maintain good health, including the identification of symptoms and the ability to understand and evaluate health-related information effectively. More specifically, MH literacy has been defined as “understanding how to obtain and maintain positive MH; understanding mental disorders and their treatments; decreasing stigma related to mental disorders; and enhancing help-seeking efficacy” [9]. Parents and caregivers are in demand of MH knowledge—especially those concerned by previous experiences with MH. However seeking help and information might be limited by fear, stigma, and mistrust of the sources of information [10]. Indeed, the lack of MH knowledge and the stigma attached to mental impairments constitute major obstacles to health care access [11]. When faced with psychiatric difficulties in their children, many parents express a lack of knowledge and the necessary skills to address these issues effectively [12]. Consequently, almost all parents (96%) turn to online sources to find answers to health-related questions in general, with MH and parenting being the most frequently searched topics [13]. However, a study examining French-speaking MH websites conducted by Buteau-Poulin et al [14] revealed that only 43% of dedicated websites provided scientifically reliable content. Among these websites, only 42% addressed autism, 45% addressed learning disorders, and merely 10% covered language disorders and behavioral problems. Furthermore, MH information websites often employ language that exceeds the average parent’s comprehension level [15].

Better functional and communicative health literacy was associated with reduced stigma and reluctance to seek MH support among adults [16]. Digital health interventions in particular have the potential to be an effective—and cost-efficient—method for increasing parental knowledge regarding MH difficulties in their children and adolescents. These interventions can assist parents in assessing valid information and understanding the organization of psychiatric services [17]. In line with this goal of supporting children and their families, we launched a website in March 2020, intended for families of children with MH disorders in the context of the first French lockdown during the COVID-19 pandemic. This website provides evidence-based knowledge on MH topics, practical tool kits, and know-how to deal with children’s everyday difficulties. CléPsy is divided into categories (general information, anxiety and affective disorders, attention-deficit/hyperactivity disorder [ADHD], intellectual development disorder, addictology, autism spectrum disorders, language/learning disorders, and eating disorders). Examples of pages include information about augmentative and adaptive communication for children with autism spectrum disorders and how to use it, what early signs of ADHD should parents be aware of, or the mechanisms and risk factors of eating disorders. Authors are identified on each page and sources are mentioned when needed.

The main purpose of this study was to evaluate users’ general acceptance of the design and the content of CléPsy, a website dedicated to helping families concerned with child MH through information and practical tools. A secondary objective was to evaluate the effect of users’ characteristics on general acceptance.

## Methods

### Creation of the Online Platform

The first version of the website [18] was created and released in March 2020 by the authors of this paper as a rapid response to the MH concerns raised by families in the context of the COVID-19 crisis. A later version (rebranded as CléPsy [19]) was designed by a team of professional web developers and made available as of January 2021. The developer team sought general feedback from families and other website users to guide their strategy but did not implement specific focus groups. Since then, the platform has offered varied content covering a large array of MH topics in French. The content of the website was produced by the multidisciplinary staff of the Center of Excellence for Neurodevelopmental Disorders (Robert Debré Hospital, Paris), submitted to a proofreading committee, and had to follow specific editorial standards based on the eEurope 2002 Quality Criteria for Health Related Websites [20].

### Questionnaires and Study Participants

A self-directed survey was made available through various methods between May and July 2022. First, the survey was directly sent to the CléPsy mailing list newsletter subscribers, which is usually used to give information about new content. Second, the survey was shared on social networks (Facebook, Twitter, and LinkedIn). Finally, posters with a QR code leading to the survey were displayed in Robert Debré Hospital. This web-based survey verified the recommendations of CHERRIES (Checklist for Reporting Results of Internet E-Surveys) for web survey quality [21] and was adapted from the questionnaire developed by Tlach et al [22] for the German e-portal Psychenet [23] and translated to French. The open survey was displayed on Lime Survey, and information about the aim and length of the survey was given before participants were asked if they consented to answer. No incentive was used to promote answering, and IP addresses were used to avoid multiple responses by the same visitor. The questionnaire first explores the baseline characteristics of respondents: age, gender, location, and educational level, as well as respondents’ experience with MH. In a second acceptance/usability–related section, we used a 4-point Likert scale (1=disagree, 2=somewhat disagree, 3=somewhat agree, 4=agree) to explore four dimensions: “Perceived ease of use,” which refers to the readability, design, and accessibility of the website; “Perceived usefulness,” which refers more specifically to the content and how it helped participants; “Perceived trustworthiness,” which is about how much the information on the website seems trustworthy and up to date; and finally, “general attitude toward the website” had questions about the tendency to recommend the website to others or to revisit it.

### Web Analytics

Google Analytics was used to determine the number of connection sessions since the launch of the website as well as demographic information about the visitors, including the countries and cities associated with the users, the frequency and recency of the sessions, and the top site contents viewed. Google Analytics is a widely used web analytics service offered by Google that tracks and reports website traffic.

### Data Analysis

The collected data on user characteristics and acceptance were analyzed on JMP statistical software (V17.0; JMP Statistical Discovery LLC). Our analysis was based on a similar work conducted by Tlach et al [22]. Only complete answers were analyzed. Means, SDs, and frequency distributions were calculated for each item to quantify the response. A total score for each of the four dimensions was calculated by summing the Likert score of all items in this dimension. One-way ANOVAs were conducted for interval-scaled variables (total scores of the four dimensions of acceptance and usability) to explore the effects of distinct participant characteristics (gender, age, educational level, experience with mental disorders, frequency of use) on the acceptance and usability of the website. A *P* value <.05 was considered to be significant for all analyses. The significance level was not adjusted as the tests served to generate hypotheses.

### Ethical Considerations

This study was submitted to and approved by the local Assistance Publique - Hopitaux de Paris ethics board (2021‐588).

## Results

### Overview

From May to July 2022, 53,911 pages were visited on CléPsy by 20,513 independent visitors. Among them, 693 (3.4%) accessed the web-based questionnaire and 614 (3%) consented to participate. Among these 614 respondents, half responded completely (n=317, 51.6%), of which almost all were female (n=297, 94%) with an average age of 46.2 (SD 10.1) years. We observed that 63.1% (n=200) of the complete respondents were MH professionals and 65.6% (n=208) were highly educated (master’s degree or a doctorate). Almost all respondents used the internet daily (n=298, 94%), and 49.2% (n=156) learned about CléPsy through social networks. Finally, 60.6% (n=192) of respondents visited CléPsy >5 times (Table 1).

Participants’ responses to the four dimensions are summarized in Table 2.

**Table 1.** Descriptive characteristics of access paths and website use (n=317).

Variable		Participants	
Gender (female), n (%)		297 (94.0)	
**Age**			
	Participants, n (%)	297 (94.0)	
	Years, mean (SD)	46.2 (10.1)	
**Education level, n (%)**			
	GCSE^a^	2 (0.6)	
	IB^b^ level	15 (4.7)	
	Undergraduate education	7 (2.2)	
	Bachelor’s degree	85 (26.9)	
	Master’s degree	149 (47.0)	
	Doctorate degree	59 (18.6)	
**Experience with mental disorders, n (%)**			
	Professional	200 (63.0)	
	Family	88 (27.8)	
	Individually	10 (3.2)	
	Other	13 (4.1)	
	Not concerned	6 (1.9)	
**Internet usage, n (%)**			
	(Almost) every day	298 (94.0)	
	At least once a week	7 (2.2)	
	At least once a month	11 (3.5)	
	Almost never	1 (0.3)	
**Access to portal, n (%)**			
	Directly	60 (18.9)	
	Via search engine	97 (30.6)	
	Via referring website	160 (50.5)	
**Awareness of the portal through, n (%)**			
	Poster	5 (1.6)	
	Recommendation from a health professional	75 (23.7)	
	Via social networks	156 (49.2)	
	Word of mouth	18 (5.7)	
	Press	8 (2.5)	
	Other	55 (17.3)	
**How many times have you been on CléPsy, n (%)**			
	Only once	56 (17.6)	
	<5	69 (21.8)	
	≥5	192 (60.6)	

a GCSE: General Certificate of Secondary Education.

b IB: International Baccalaureate.

**Table 2.** User rating on perceived ease of use, perceived usefulness, attitude toward using the portal, and perceived trust (n=317).

Variables		Agree, n (%)	Somewhat agree, n (%)	Somewhat disagree, n (%)	Disagree, n (%)
**Perceived ease of use**					
	The font of the website is easy to read	176 (55.5)	129 (40.7)	8 (2.5)	4 (1.3)
	The website is easy to use	152 (48.0)	152 (48.0)	8 (2.5)	5 (1.5)
	The presentation of the information is clearly arranged	141 (44.5)	151 (47.6)	19 (6.0)	6 (1.9)
	The information is easy to understand	184 (58.0)	121 (38.2)	8 (2.5)	4 (1.3)
	The design of the website is appealing	146 (46.1)	158 (49.8)	10 (3.2)	3 (0.9)
	The colors of the website are pleasant	168 (53.0)	132 (41.7)	15 (4.7)	2 (0.6)
	The pictures on the website are appropriate	143 (45.1)	159 (50.2)	13 (4.1)	2 (0.6)
	I can quickly find the information that is important to me	124 (39.1)	162 (51.1)	28 (8.9)	3 (0.9)
**Perceived usefulness**					
	The content of the website is interesting	224 (70.7)	87 (27.4)	2 (0.6)	4 (1.3)
	All in all, the website is useful for me	196 (61.8)	112 (35.3)	5 (1.6)	4 (1.3)
	The amount of information presented on the website is appropriate	158 (49.8)	138 (43.5)	17 (5.4)	4 (1.3)
	The website contains information that I need	157 (49.5)	141 (44.5)	17 (5.4)	2 (0.6)
	The information on the website has helped me with my concerns	152 (48.0)	148 (46.7)	14 (4.4)	3 (0.9)
	Through the website, I received references to other sources	114 (36.0)	160 (50.5)	41 (13.0)	2 (0.6)
	Through the website, I learned something new	145 (45.7)	145 (45.7)	23 (7.3)	4 (1.3)
	Now I’m able to talk better about mental disorders with my health professional	105 (33.1)	159 (50.2)	51 (16.1)	2 (0.6)
	Now I’m able to talk better about mental disorders with my relatives	101 (31.9)	159 (50.2)	54 (17.0)	3 (0.9)
**Attitude toward using**					
	I would recommend the website to others	232 (73.2)	76 (24.0)	5 (1.6)	4 (1.2)
	I will revisit the website if needed	249 (78.5)	62 (19.6)	2 (0.6)	4 (1.3)
**Perceived trust**					
	The information on the website is trustworthy	231 (72.9)	77 (24.3)	6 (1.9)	3 (0.9)
	The information on the website is up to date	184 (58.0)	120 (37.9)	10 (3.2)	3 (0.9)

### Perceived Ease of Use

Almost all participants (n=304, 96%) stated that the website was easy to use, with 90.2% (n=305) indicating that they could easily find the information important to them. Additionally, 96% (n=304) found the design appealing. None of the main participants’ characteristics had a specific effect on the perceived ease of use (Table 3).

**Table 3.** Effects of participants’ characteristics on the perceived ease of use (n=317).

		Participants, n	Perceived ease of use score		*F* test (*df*)	*P* value
			Mean	SD		
**Gender**					0.97 (1)	.33
	Female	297	6.77	0.06		
	Male	20	6.52	0.24		
**Age (years)**					1.93 (1)	.16
	≤46	172	6.67	0.08		
	>46	144	6.84	0.09		
**Educational level**					1.44 (6)	.20
	GCSE^a^	2	7.37	0.76		
	IB^b^ level	15	6.58	0.28		
	Undergraduate degree	7	6.68	0.40		
	Bachelor’s degree	85	6.62	0.12		
	Master’s degree	149	6.77	0.09		
	Doctorate degree	59	6.93	0.14		
**Experience with mental health**					0.71 (4)	.58
	Professional	200	6.82	0.07		
	Family	88	6.64	0.11		
	Individually	10	6.65	0.34		
	Other	13	6.58	0.30		
	Nonconcerned	6	6.46	0.44		
**Frequency of use**					2.44 (2)	.09
	Only once	56	6.47	0.14		
	<5	69	6.79	0.13		
	≥5	192	6.82	0.08		

a GCSE: General Certificate of Secondary Education.

b IB: International Baccalaureate.

### Perceived Usefulness

The majority of respondents agreed or somewhat agreed with the statement “All in all, the website is useful for me” (n=308, 97.1%). A significant percentage agreed or somewhat agreed that the website facilitated discussions about MH with professionals (n=264, 83.3%) or with their relatives (n=260, 82.1%). According to the ANOVA, there was a significant effect between frequency of use and perceived usefulness (*F*_2_=4.85; *P*=.008; Table 4).

**Table 4.** Effects of participants’ characteristics on the perceived usefulness (n=317).

			Participants, n	Perceived usefulness score		*F* test (*df*)	*P* values
				Mean	SD		
**Gender**						0.01 (1)	.91
	Female		297	7.60	0.06		
	Male		20	7.62	0.25		
**Age (years)**						0.15 (1)	.71
	≤46		172	7.62	0.08		
	>46		144	7.57	0.09		
**Educational level**						0.22 (6)	.95
	GCSE^a^		2	8.25	0.80		
	IB^b^ level		15	7.48	0.29		
	Undergraduate degree		7	7.39	0.42		
	Bachelor’s degree		85	7.62	0.12		
	Master’s degree		149	7.60	0.09		
	Doctorate degree		59	7.60	0.15		
**Experience with mental health**						0.33 (4)	.86
	Professional		200	7.62	0.08		
	Family		88	7.56	0.12		
	Individually		10	7.77	0.35		
	Other		13	7.54	0.31		
	Nonconcerned		6	7.17	0.46		
**Frequency of use**						4.85 (2)	.008
	Only once		56	7.33	0.15		
	<5		69	7.38	0.13		
	≥5		192	7.75	0.08		

a GCSE: General Certificate of Secondary Education.

b IB: International Baccalaureate.

### Perceived Trust and General Attitude Towards Using CléPsy

The vast majority of respondents described the website as trustworthy (n=308, 97.3%) and up to date (n=304, 95.9%), and would recommend it to other people (n=308, 97.2%). None of the main participants’ characteristics had a specific effect on their attitude toward using CléPsy (Table 5). According to the ANOVA, there was a significant effect relating perceived trust with the educational level (*F*_6_=3.03; *P*=.007) and the frequency of use (*F*_2_=5.30; *P=*.006; Table 6).

**Table 5.** Effects of participants’ characteristics on attitudes toward the website (n=317).

		Participants, n	Attitudes toward the website score		*F* test (*df*)	*P* value
			Mean	SD		
**Gender**					0.44 (1)	.50
	Female	297	3.42	0.02		
	Male	20	3.47	0.07		
**Age (years)**					0.46 (1)	.50
	≤46	172	3.44	0.02		
	>46	144	3.41	0.02		
**Educational level**					0.64 (6)	.70
	GCSE^a^	2	3.75	0.23		
	IB^b^ level	15	3.43	0.08		
	Undergraduate degree	7	3.36	0.12		
	Bachelor’s degree	85	3.40	0.03		
	Master’s degree	149	3.43	0.03		
	Doctorate degree	59	3.45	0.04		
**Experience with mental health**					0.32 (4)	.86
	Professional	200	3.43	0.02		
	Family	88	3.40	0.03		
	Individually	10	3.45	0.20		
	Other	13	3.46	0.09		
	Nonconcerned	6	3.50	0.13		
**Frequency of use**					0.09 (2)	.91
	Only once	56	3.41	0.04		
	<5	69	3.43	0.04		
	≥5	192	3.43	0.02		

a GCSE: General Certificate of Secondary Education.

b IB: International Baccalaureate.

**Table 6.** Effects of participants’ characteristics on the perceived trust (n=317).

		Participants, n	Perceived trust score		*F* test (*df*)	*P* value
			Mean	SD		
**Gender**					0.09 (1)	.76
	Female	297	3.61	0.03		
	Male	20	3.57	0.12		
**Age (years)**					0.09 (1)	.76
	≤46	172	3.60	0.04		
	>46	144	3.62	0.04		
**Educational level**					3.03 (6)	.007
	GCSE^a^	2	2.50	0.38		
	IB^b^ level	15	3.37	0.20		
	Undergraduate degree	7	3.64	0.20		
	Bachelor‘s degree	85	3.53	0.06		
	Master’s degree	149	3.650	0.04		
	Doctorate degree	59	3.72	0.07		
**Experience with mental health**					0.31 (4)	.87
	Professional	200	3.62	0.04		
	Family	88	3.61	0.06		
	Individually	10	3.55	0.17		
	Other	13	3.46	0.15		
	Nonconcerned	6	3.58	0.22		
**Frequency of use**					5.30 (2)	.006
	Only once	56	3.41	0.07		
	<5	69	3.59	0.06		
	≥5	192	3.67	0.04		

a GCSE: General Certificate of Secondary Education.

b IB: International Baccalaureate.

## Discussion

### Principal Findings

In this web-based study, we investigated the acceptance of the design and content of a new website dedicated to children’s MH information with the aim of gaining a better understanding of active users and areas for improvement to reach target users more effectively.

Among complete responders (n=317), almost all agreed (n=152, 47.9%) or somewhat agreed (n=152, 47.9%) with the affirmation that the website was easy to use. Those results were in line with previous studies exploring the ergonomics of online MH information. When evaluating the Psychenet website, Tlach et al [22] found equivalent rates of perceived ease of use. Engagement and perceived ease of use were equivalent regarding the Together for Wellness website, developed during the COVID-19 pandemic [24]. A preference for English and higher behavioral changes during the pandemic were predictors for higher comfort in using the Together for Wellness website. Concerning the design of the CléPsy website, most participants evaluated the font, the colors, the presentation of information, the illustrations, and the accessibility of information positively. User experience (UX) is an important feature of online MH information, and it can be a major barrier for end users [25]. In our study, perceived ease of use did not differ with participants’ characteristics. This underlines the importance of working with UX and design professionals to create state-of-the-art internet navigation interfaces that are easy to use. This is especially true when implementing online health information.

The usefulness of the CléPsy website was largely appreciated by the respondents. This could be in line with an almost complete lack of accessible digital information about child and adolescent MH written in French. Furthermore, two-thirds of French people believe that digital tools can help them better understand health care strategies [26]. This is especially the case for MH information, which is one of the main topics researched by parents online when they are looking for pediatric health information [13]. Parents could also be more aware of their children’s difficulties with the increasing awareness of MH issues in children and adolescents, especially since the COVID-19 pandemic [27]. For example, the online search for ADHD information has been increasing for several years, which could partially be explained by destigmatization induced by media coverage [28]. The perceived usefulness had a positive influence on the user’s ability to discuss MH with health professionals (including their general practitioners) or family members. Indeed, as reported in a systematic review by Kubb and Foran [29], most parents expressed difficulties in talking to their physicians about health information found online. The main reasons expressed by parents for not talking to the physicians were a lack of time, a fear of disapproval, or difficulties in understanding technical medical terminology. Interestingly, physicians express reservations on the subject. In a qualitative study, Karatas et al [30] explored the main attitude of pediatricians confronted with eHealth-based questions. Most of them expressed concerns about inaccurate health information that could lead to detrimental decisions by families. They highlighted an increased length of consultations to answer the questions but acknowledged that it helped build a more collaborative relationship with parents. In our study, most respondents were MH professionals. This could reflect a need for regular updates on practices, especially regarding evidence-based interventions, even if the therapist’s behavior seems to change not only with information but also with specific training [31]. A quantitative study confirmed the positive and bidirectional influence of the perceived usefulness of eHealth information on patient-physician interaction and trust [32]. From the patient’s perspective, it enriches participation and cooperation through enhanced resource integration, and from the physician’s perspective, it can improve the patient’s understanding and the relationship.

The CléPsy website was considered trustworthy by 97.2% (n=308) of respondents, but ANOVAs found an effect of educational level on the perceived trust, contrary to the evaluation of Psychenet by Tlach et al [22]. Parents generally have difficulties evaluating the trustworthiness of their source of information when searching for child health information [33], but most of them would like health care professionals to help them identify reliable sources [34]. When parents from Switzerland were asked about their relationship to online health information seeking, most of them were skeptical about the correctness of the information, but one-third of them did not, or rarely, check the reliability of the website [35]. Interestingly, first-time parents and parents with high levels of education made higher use of web pages targeted at parents. Trust in online health information is predicted by sociodemographic factors such as higher income and educational level [36], but online information remained the least trusted source, behind media sources and interpersonal information.

### Limitations

This study must be considered with regard to several limitations. First, contrary to our expectations regarding the main audience, the majority of respondents were MH professionals, whereas the website was created to address inquiries from families. Furthermore, a significant proportion of respondents had a high level of education (master’s or doctorate). This may be attributed to the distribution method of the questionnaire, in particular via professional social networks, and to the institutional credibility of the Center of Excellence for Neurodevelopmental Disorders in France. In our analysis, the characteristics of the respondents did not have any effect on their answers. Considering the accessibility and usability of health websites, the literature shows that primary users and MH professionals tend to have the same opinion [37]. MH professionals could also be interested in our website due to the necessity to get valid information about the state-of-the-art practice in MH. Indeed, an important part of the French psychology curriculum is dedicated to psychoanalysis. Future studies would need to consider the differences between these audiences. Moreover, the representativeness of the respondent must be interpreted with caution as the response rate was very low compared to the total number of users during the same period. Second, the CléPsy website was created initially to address families’ questions about their children’s MH at the onset of the COVID-19 pandemic and facilitate the transmission of relevant content. However, due to this unique timeline, the current version of the website insufficiently meets the standard framework that recommends the co-construction of health information websites together with families and other users [38]. Focus groups with families have since been held by our team along with the UX professionals, resulting in a qualitative evaluation of parents’ needs. Third, as expressed by Tlach et al [22], there is a lack of standardized instruments to evaluate the acceptability and usefulness of digital content, which limits comparison. However, the questionnaire was designed based on recent recommendations such as the CHERRIES checklist. Finally, adverse effects of online health information seeking such as cyberchondria were not evaluated and should be taken into account in further research [39].

### Conclusion

Our study provides preliminary support for the acceptability and usefulness of a French MH information website dedicated to children and adolescents. Results show that users are in demand of trustworthy information on this matter. In the future, special attention should be given to the co-construction of such websites with concerned users to improve the pertinence, inclusiveness, and accessibility of the contents to a wide and varied public. Moreover, parental role and child diagnosis should be identified in future research.

## Abbreviations

ADHD: attention-deficit/hyperactivity disorder
CHERRIES: Checklist for Reporting Results of Internet E-Surveys
MH: mental health
UX: user experience
